# Referential and inferential production across the lifespan: different patterns and different predictive cognitive factors

**DOI:** 10.3389/fpsyg.2023.1237523

**Published:** 2023-10-30

**Authors:** Raphaël Fargier, Marina Laganaro

**Affiliations:** ^1^Université Côte d'Azur, CNRS, BCL, Nice, France; ^2^Neuropsycholinguistics Laboratory, Faculty of Psychology and Educational Sciences, University of Geneva, Geneva, Switzerland

**Keywords:** language production, lifespan, referential picture naming, inferential naming from definitions, aging

## Abstract

**Introduction:**

The ability to speak is grounded in general memory and control processes and likely changes across the lifespan. However, our knowledge on how word production abilities naturally evolve from childhood to old age remains marginally investigated. Our aim was to shed further light on this issue by exploiting the contrast between two ways to elicit word production: referential picture naming and inferential naming from definition.

**Methods:**

We collected accuracy and production latencies in a picture naming task and in a naming from definition task from 130 participants ranging from 10 to 80 years old. Measures of vocabulary size, digit span memory, semantic and phonemic fluencies and processing speed were also collected. We used multivariate adaptative regression splines and regression models to characterize lifespan patterns of the two tasks.

**Results:**

Patterns of increase in performance were similar for picture naming and naming from definition only from childhood to young adulthood. In the second half of the lifespan, significant decrease of performance was found in older adults for picture naming (from around 60 years-old) but not for naming from definition. Clearly, word production elicited with an inferential task (naming from definition) yields different age-related patterns than usually described in the literature with a referential task (picture naming).

**Discussion:**

We discuss how cognitive processes such as visual-conceptual processes and lexical prediction may explain the differential pattern of results in aging in referential and inferential production tasks. We argue for more lifespan studies and the need to investigate language production beyond picture naming, in particular with respect to aging.

## Introduction

Speaking is a pervasive activity in which we engage throughout our entire lives. In a nutshell, speaking requires transforming ideas into articulated speech sounds, through the selection of words in our long-term memory. The ability to speak is thus grounded in general memory and in control and executive processes, which likely display changes across the lifespan. Unfortunately, how language production abilities naturally evolve from childhood to old age remains blurry, and investigation is mostly focused on referential production tasks such as picture naming. Yet, the task itself might target specific cognitive processes that do not sum up language production. Evidence from inferential production tasks, such as naming from definition, is scarce. Our aim is thus to shed further light on the issue of changes in word production across the lifespan by exploiting the contrast between two tasks, both of which elicit word production, but do so through different cognitive processes: referential picture naming and inferential naming from definition. Using the same sample of individuals, we sought (1) to characterize similarities and differences in patterns of production elicited by pictures (referential) and definitions (inferential), from childhood to late adulthood, and (2) to identify the relative contribution of several cognitive abilities in the two tasks.

### Changes in language production abilities across the lifespan: behavioral indicators

The most striking indicators that our ability to speak evolves across a lifetime stem from picture naming and are quantitative. Word production ability is often probed with referential naming tasks, also called picture naming or confrontation naming (Levelt, 1989; Dell and O'Seaghdha, 1992; Glaser, 1992; Caramazza, 1997). Referential picture naming is eliciting a word or an utterance from a visually depicted concept. First of all, lexical learning grows continuously throughout the lifespan: People's vocabulary reaches 300 words ~2 years of age (Dale and Fenson, 1996), ~10,000 words around the age of 6 years (Clark, 1993), and up to 50,000 words in adulthood (Aitchison, 2012). Production latencies also vary throughout the lifespan and are slower at both extremes of the developmental curve. Picture naming follows a U-shape or bow-shape trend, with lower accuracy and increased production latencies in children and older adults relative to young adults (Nicholas et al., 1997; Feyereisen et al., 1998; D'Amico et al., 2001; Evrard, 2002; Morrison et al., 2003; Connor et al., 2004; Newman and German, 2005; Thornton and Light, 2006; Kavé et al., 2010; Verhaegen and Poncelet, 2013; Laganaro et al., 2015; Valente and Laganaro, 2015). Moreover, older adults often report increased tip-of-the-tongue states (TOTs), that is, increased failure to retrieve a word despite a strong feeling of knowing (Brown, 1991; Burke et al., 1991).

Despite quantitative differences in word production in children and old adults relative to (young) adults, it seems that the same cognitive processes underlie language production at all ages. For instance, the same psycholinguistic variables predict accuracy (Cycowicz et al., 1997) and production latencies (D'Amico et al., 2001) in picture naming in school-age children and adults. The results from variants of the picture naming task such as picture–word interference paradigms (PWI) also converge to similar processes being called for in speaking across the lifespan (Brooks and MacWhinney, 2000; Taylor and Burke, 2002).

### Changes in language production abilities across the lifespan: neural indicators

Some electrophysiological studies using picture naming tasks showed similar waveforms in children and adults despite differences in latencies, matching the idea of overall similar neural substrates (Greenham and Stelmack, 2001; Budd et al., 2013). However, another study revealed both functional qualitative and quantitative changes between school-age children and young adults (Laganaro et al., 2015). Qualitative changes were reflected by different global electrophysiological topographic patterns of the scalp occurring in early time windows, suggesting that pre-linguistic (visual and conceptual) processes undergo changes across development, the transition through adolescence being of critical interest for further investigations (Atanasova et al., 2020). Using a similar approach to compare neural activity in adults and older adults, Valente and Laganaro (2015) showed between-group neural differences in two time periods associated with semantic (early) and phonological (late) processes. Studies using functional magnetic resonance imaging (fMRI) reported overlapping brain activations in children and adults during word production and also areas displaying different responses (e.g., left frontal and left parietal areas, see Brown et al., 2005; Krishnan et al., 2015). Studies also looked at the effects of aging on the language neurocognitive architecture. Wierenga et al. (2008) showed, for instance, that in older individuals, the frontal cortex was more activated in language production tasks, which was associated with a similar level of accuracy (see also Kemper et al., 2001). Since there was no difference in inferior temporal areas, this effect was interpreted in favor of difficulties in retrieval processes rather than changes in long-term memory storage. Interestingly enough, although research on language aging seeks the correlates of decremental word retrieval abilities (see Burke and Shafto, 2004; Stine-Morrow et al., 2010), neuroimaging studies often report similar levels of performance between young and old adults; thus, differential brain activity likely reflects more compensatory mechanisms than decline (see Baciu et al., 2016; Hoyau et al., 2016).

### Linguistic vs. cognitive explanations of age-related changes

It is not straightforward to assume that the same factors explain language production at all ages and in all contexts of utterance production; the various processes underlying word production may undergo different life courses. On the one hand, changes in language production abilities may mirror changes in general information processing speed (Myerson et al., 1990, 1992; Salthouse, 1996), executive functions (Craik and Byrd, 1982), or inhibitory functions (Hasher et al., 2007; Lustig et al., 2007) like those involved in selecting a target word among competitors (Astell and Harley, 1996). These cognitive abilities mature across development and decline with age, likely contributing to the early increase and late decrease of word retrieval abilities (Murphy et al., 2000; Burke and Shafto, 2004). On the other hand, age-related increase and decrease in performance in word retrieval could relate to changes in specific components of word retrieval such as the size, structure, and organization of the mental lexicon (Dubossarsky et al., 2017; Wulff et al., 2019). The architecture of the mental lexicon (e.g., number of lexical representations and the strength of relationships) likely constrains lexical access, and it was proposed that changes, for example, due to accumulated learning, could explain some of the apparent decremental effects on word retrieval seen in old adults (Ramscar et al., 2014; Wulff et al., 2019) and explain age-specific performance in language production (Krethlow et al., 2020). Accordingly, the vocabulary of individuals, whether high or low, would be a better predictor of performance in old adults than cognitive decline *per se*.

According to the Transmission Deficit Hypothesis (TDH) (Burke and Shafto, 2004), older adults display a worse performance in word production (decreased accuracy, slower latencies, and increased TOTs) because of the weakening of connections among linguistic representations, leading to reduced transmission of excitation from one representation to another. This is assumed to occur specifically at the lexical-phonological level because older adults have intact and redundant semantic representations and processing, which are not/less affected by weakening of connections, but can fail to activate phonological representations.

Yet, the cognitive and linguistic factors that characterize changes in word encoding processes across the lifespan remain unclear. Coherent with that, Boudiaf et al. (2018) attempted to disentangle the effects of cognitive and linguistic factors on word retrieval decline in aging. They gathered performance in various linguistic and non-linguistic tasks including picture naming, categorization, or numerical and color judgment, in participants aged between 30 and 84 years of age. They showed that production latencies in picture naming become faster with age, once general processing speed effects are parceled out, suggesting increased automaticity in referential production. Yet, it may simply reflect that the decremental effects of aging on processing speed do not match the decremental effects of aging on referential naming. Although this study heads in the right direction, research must examine the effects of abilities such as vocabulary, working memory, fluency and processing speed on other language production tasks, and span larger age ranges.

### Beyond referential naming

As mentioned so far, picture naming has been extensively used as a proxy to study language production abilities, develop theories about the cognitive architecture of word production in healthy and pathological conditions, and in development and aging as well (Indefrey and Levelt, 2004). However, confrontation naming contexts are not the only ways word production is triggered in real life, and the mechanisms supporting *inferential* production ability, i.e., the production of utterances via semantic and episodic associations, are far less known.

Referential naming and inferential naming share important features as they both are required to select a word from long-term memory and transform it into articulated speech sounds. Accordingly, they share similar neural substrates (Marconi et al., 2013), and word-form encoding, in particular, is achieved through similar processes in both tasks (Fargier and Laganaro, 2017). However, both tasks also differ in one important respect, which is *how* the word is selected: from a visual concept in picture naming and through semantic and/or episodic associations in inferential naming. Fargier and Laganaro (2017) tested this assumption by recording electroencephalographic activity while participants were producing words from pictures or from written definitions (e.g., “Animal from which we obtain honey” *bee*, “Object that we use to shoot arrows” *bow*). They examined the underlying patterns of neural activity and showed that the early processes underlying production (<400 ms) had different topographic configurations for the two tasks, e.g., different neural microstates. Precisely, they found a left-lateralized frontal positivity for naming from definition and a right-lateralized posterior positivity for picture naming. As each microstate is known to represent different active neural networks (Koenig et al., 1998), the authors suggested that early processes were achieved through different neural networks in the two tasks. Moreover, they performed additional analyses, in which psycholinguistic predictors of each word were used to show that semantic processes (i.e., evidenced by the level of animacy of words) clearly preceded lexical processes (i.e., evidenced by word frequency) in naming from definition, while the opposite was found in picture naming. Considering that lexical frequency effects traditionally mark the onset of lexical selection, the authors suggested that early lexical and semantic processes were achieved through different cognitive and neural operations in naming from definition and picture naming (Fargier and Laganaro, 2017; see also Calzavarini, 2017; Atanasova and Laganaro, 2022).

Obviously, most models of lexical retrieval propose that in picture naming, the visual input activates semantics (Levelt et al., 1999). Yet, picture naming is constrained by the visuo-conceptual input (see Fargier and Laganaro, 2015), and it is unclear whether the depth of semantic activation (e.g., the activation of additional non-visual information) is equivalent to what should be observed in naming from definition. The issue of the depth of semantic processes in picture naming tasks has been raised previously by authors suggesting that naming pictures may be achieved with minimal semantic processes (Heilman et al., 1976; Kremin, 1986, 1988; Silveri and Colosimo, 1995; Brennen et al., 1996). In naming from definition paradigms, for instance, the speaker has to produce a word in response to a given written or oral definition, thus the information contained in the definition must be encoded and combined to retrieve the target word. This suggests that the ability to maintain the different parts of the definition in short-term memory could predict performance in naming from definition. We can hypothesize that because definitions unfold over time, individuals are likely to make online lexical predictions, which could in turn contribute to facilitating target word retrieval. In the best-case scenario, competing candidates could be eliminated early, and target words could be retrieved more accurately and/or faster. This relates to verbal and executive control abilities, in particular, those involved in semantic fluency (see Shao et al., 2014), which requires selecting and managing items from competing targets in semantic categories (Thompson-Schill et al., 1997). Altogether, we assume that comprehension, working memory, and control processes may be more involved in word retrieval from verbal definitions, whereas visuo-conceptual processes would rather drive word retrieval in picture naming. The underlying hypothesis that the two tasks rely on different weights of cognitive abilities allows us to assume that word retrieval will not display the same trajectory across the lifespan, in particular, in late adulthood.

As mentioned earlier, TOTs are more frequent in older adults compared to young adults, and this can be observed both with referential naming, such as object naming or naming famous people's faces, or inferential naming (from definitions or descriptions) (e.g., Burke et al., 1991; Brown and Nix, 1996; Salthouse and Mandell, 2013; see Burke and Shafto, 2004 and Brown, 2011 for reviews). In their study, Salthouse and Mandell (2013) found a higher frequency of TOTs in older adults compared to young people in tasks that involved pictures or descriptions of famous people but not in tasks where the words had to be found in response to definitions. The sensitivity to different kinds of materials might reflect the discrepancy in TOTs across experiments and populations, and it remains unclear whether visual-conceptual to phonological representations are more likely to weaken in aging compared to routes from semantic associations to phonological representations. Note that naming from definition has been used in clinical practice for a long time as well and in the assessment of language production in epilepsy (Sartori and Lombardi, 2004; Trebuchon-Da Fonseca et al., 2009), hence characterizing the patterns across the lifespan of both referential picture naming and inferential naming from definitions continues to be of paramount importance for clinical perspectives.

### Our goals

Given that different word production contexts likely involve different cognitive abilities, here, we take advantage of the contrast between picture naming and naming from definition to characterize the patterns of word production from childhood to late adulthood and to identify underlying cognitive abilities. Participants aged from 10 to 80 years underwent both picture naming task and naming from definition task and were administered several other cognitive tasks. We considered different cognitive processes involved in word production such as long-term memory (vocabulary breadth), short-term memory (digit span), executive control processes (fluencies), and speed (processing speed).

Our analysis consisted first of a descriptive approach using multivariate regression and second of multiple regression models to determine the factors explaining the performance at all ages.

Our hypotheses were that:

- Picture naming (referential naming) and naming from definition (inferential naming) do not display the same patterns across the lifespan because they do not rely entirely on the same cognitive processes. Specifically, as naming from definition relies more on semantic processes, which are assumed to be preserved in aging, performance in naming from definition should not decline as much as in picture naming in older adults.
- Despite obvious similarities, word retrieval in picture naming and naming from definition does not rely entirely on the same cognitive processes. Specifically, performance in naming from definition may be predicted by vocabulary breath and working memory to a larger extent than picture naming.

## Materials and methods

### Participants

A total of 142 neurotypical participants ranging from 10 to 80 years of age took part in the study. Because of unfortunate data loss in one or another experimental task, the analyses were performed on 130 participants for which data were complete in all experimental tasks. Participants were spread across six defined age groups including children of 10–13 years of age (*N* = 19, mean age = 11 ± 0.8; 8 female participants) and adolescents of 15–18 years of age (*N* = 24, mean age = 16.7 ± 1; 14 female participants), as well as groups of adults with an age range of 1 decade, including young adults of 20–30 years of age (*N* = 23, mean age = 24.7 ± 3; 13 female participants), middle-aged adults of 40–50 years of age (*N* = 17, mean age = 45.6 ± 3.5; 10 female participants), senior adults of 58–68 years of age (*N* = 26, mean age = 64.1 + 3.2; 16 female participants), and old adults >70 years of age (*N* = 21, mean age = 73.3 ± 3.1; 14 female participants). Participants were French native speakers, right-handed, and had normal or corrected-to-normal vision. None reported a significant history of language, psychiatric, or neurological impairments. The final dataset comprised 130 participants for whom all data were collected (see Results section). Years of education was recorded as additional demographic information, although it was not helpful for the younger groups of children and adolescents, as they were all in the same class. On average, the number of years of education was 7.4 (±1.2) in children, 11.2 (±1.2) in adolescents, 16.7 (±2.2) in young adults, 17.3 (±4.4) in middle-aged adults, 14.2 (±3) in senior adults, and 13.5 (±2.9) in the group of old adults. Participants were recruited in the area of Geneva in Switzerland. This is known to be a multicultural area, thus participants usually speak multiple languages. We made sure their native language was French and asked participants whether they spoke other languages, but we did not investigate further language experience as it was beyond the scope of our study.

The study was approved by the local ethics committee of the Faculty of Psychology and Educational Science of the University of Geneva, and all participants gave their written informed consent before the study. Note that for children and adolescents under the legal age, parents' approvals were collected. All participants were paid for their participation.

### Tasks

Different tasks were conducted, including two language production tasks (referential and inferential naming tasks) and two tasks measuring processing speed (Simple and Forced choice Reaction Time tasks). These tasks were computerized. Semantic and phonemic fluencies, measures of vocabulary size, and digit span memory, as well as a home-made reading task, were also included.

#### Picture naming

##### Stimuli

For referential naming, we used a picture naming task. A total of 120 black and white drawings and their corresponding modal names were selected from two French databases (Alario and Ferrand, 1999; Bonin et al., 2003). The 120 words were all acquired before the age of 9 years to ensure that they were known by the youngest participants. The word age-of-acquisition range was 1.19–3.55 on a 5-point scale (1: learned between 0 and 3 years; 4: learned between 9 and 12 years; 5: learned after the age of 12). There were 40 monosyllabic, 60 bisyllabic, and 20 trisyllabic words. Word frequency ranged from 0.13 to 227 occurrences per million words (mean = 17.3) according to the French database Lexique (New et al., 2004). All picture words had high name agreement (mean = 92.5%) (Alario and Ferrand, 1999).

#### Naming from definition

##### Stimuli

For inferential naming, we designed a naming from definition task. A total of 108 target words were selected, and their corresponding definitions were constructed similarly to previous study (Fargier and Laganaro, 2017), i.e., definitions had a similar structure across items and target words could not be anticipated before the last word of the definition was heard (e.g., “Animal from which we obtain honey” *bee*, “Object that we use to shoot arrows” *bow*). Only imageable concrete words were used, and 24 items were also used in the picture naming. All target words corresponded to words learned before the age of 9 years, according to the previously mentioned databases, and the word age-of-acquisition range was 1.27–3.65 on the 5-point scale. There were 25 monosyllabic, 65 bisyllabic, 15 trisyllabic, and 3 quadrisyllabic words. Lexical frequency ranged from 0.06 to 605.75 occurrences per million words (mean = 35.4) according to the French database Lexique (New et al., 2004).

The obtained name agreement for the definitions was on average 89%.^1^ Given that the average name agreement was 92.5% for picture naming, the level of difficulty of trials used in the inferential naming task and the referential naming task was considered equivalent. Definitions were presented orally from a female French native speaker and delivered through speakers.

#### Other tasks

Vocabulary is known to increase throughout the lifespan. We thus performed a vocabulary test taken from the Wechsler Adult/Children Intelligence Scale, Wechsler, 2005. Working memory is also known to change across the lifespan, and peaks at 20 years of age (Sander et al., 2012). To assess working memory, we used a digit span test, also taken from the Wechsler Adult/Children Intelligence Scale, Wechsler, 2005. Both tests were scored according to age standards. Two verbal fluency tasks were performed and consisted of providing within 2 min the maximal number of names from the category of animals (i.e., semantic fluency task) and of words starting with the letter P (i.e., phonemic fluency task). Repetitions had to be avoided and were excluded from the final score. The final score was the number of names provided in 2 min.

To assess processing speed, which is known to show age-related changes, we designed a simple reaction time experiment and a forced choice reaction time experiment. The simple reaction time experiment consisted of hitting, as fast as possible, a specific key on the keyboard when a cross appeared on the screen. In the forced choice reaction time experiment, two stimuli appeared simultaneously on the screen: a short and a large bar. The task consisted of hitting the key “D” when the longer bar appeared on the left side of the screen and the key “L” when it appeared on the right side. Both tasks included 120 trials.

### Procedure

The general procedure was split into two sessions. One session of “behavioral assessment” comprised the questionnaire of demographic information, the vocabulary size measure, the digit span memory task, the semantic and phonemic fluencies, the processing speed tasks, and the reading task. This order of tasks was kept constant across participants within this session. The other session with “neuroimaging recordings” included the two language production tasks and a Stroop task during which electroencephalographic activity was recorded. The results from the Stroop task can be found in Ménétré and Laganaro (2019). Electroencephalographic data on picture naming in children, adolescents, and young adults have been published elsewhere (Atanasova et al., 2020). Other neuroimaging data are not reported. The two sessions “behavioral assessment” and “neuroimaging recordings” were conducted one after the other for a combined average duration of 2 h and a half. The order of the sessions could differ across participants. Within the “neuroimaging recordings” session, the order of the tasks was controlled: either the session started with the picture naming or the naming from definition task, and the Stroop task was always in between.

#### Picture naming

Stimuli were presented on-screen: Each trial started with a centered fixation cross (500 ms) followed by the picture for 3,000 ms. Participants had to overtly produce the word corresponding to the picture as fast and as accurately as possible. The interval between the stimuli was set at 2,000 ms. Four warm-up filler trials were used at the beginning of the experiment and after the break, which occurred halfway through the task. Two pseudo-random orders were used (one pseudo-random order and its reverse order), and the task was implemented with the software E-Prime (Version 2.0) (Schneider et al., 2002).

#### Naming from definition

Each trial started with a centered fixation cross (500 ms), followed by the presentation of the oral definition. During the presentation of the definition, an exclamation point was present on the screen. Participants were instructed to overtly produce the word corresponding to the definition as fast and as accurately as possible. The interval between the stimuli was set at 2,000 ms, and five warm-up filler trials were used at the beginning of the experiment. The task was implemented with E-Prime (Version 2.0) (Schneider et al., 2002).

### Data pre-analyses

Word productions in the picture naming and the naming from definition tasks were digitized and accuracy and production latencies (reaction times in milliseconds) were systematically checked offline with a speech analysis software (Check-Vocal 2.2.6, Protopapas, 2007). In the picture naming task, the reaction time corresponded to the time separating the onset of the picture and the onset of the speech wave. In the naming from definition task, the reaction time corresponded to the time separating the offset of the last word of the definition and the onset of the speech wave. We used the phonological uniqueness point of the last word as the reference point when it differed from the offset of the last word of the definition (<5 items). For these two tasks, outliers and unexpected answers (additional articles, hesitation marks, and errors) were excluded from the RT analyses. Outliers followed a careful inspection of the data specific to each task (i.e., 500 ms < RT < 2,000 ms in referential picture naming; 250 ms < RT < 2,200 ms in inferential naming from definition).

### Data analyses

Similar analyses were performed on accuracy and production latencies for the picture naming task on the one hand and the naming from definition task on the other hand.

Two main analyses were performed. The first set of analyses consisted of characterizing the lifespan pattern of picture naming and naming from definition using multivariate adaptative regression splines (package Earth, Milborrow et al., 2013). This approach produces a regression that is allowed to bend at certain knots that mark a change in the behavior of the function. The knots obtained across the lifespan, i.e., spanning the different age groups, could then be compared between picture naming and naming from definition. The second analysis consisted of multiple regression models with several cognitive predictors to test for the critical effects of these measures on performance across the lifespan.

All these analyses were conducted with the R-software (R-project, R Core Team, 2022) and R studio (Version 0.99.903; RStudio 2009–2016).

### Transparency and openness

Data and analysis codes have been made available on the Open Science Framework and can be accessed at https://osf.io/jwyur/. This study's design and its analysis were not pre-registered.

## Results

### The effect of age on picture naming and naming from definition

**Figure 1:**
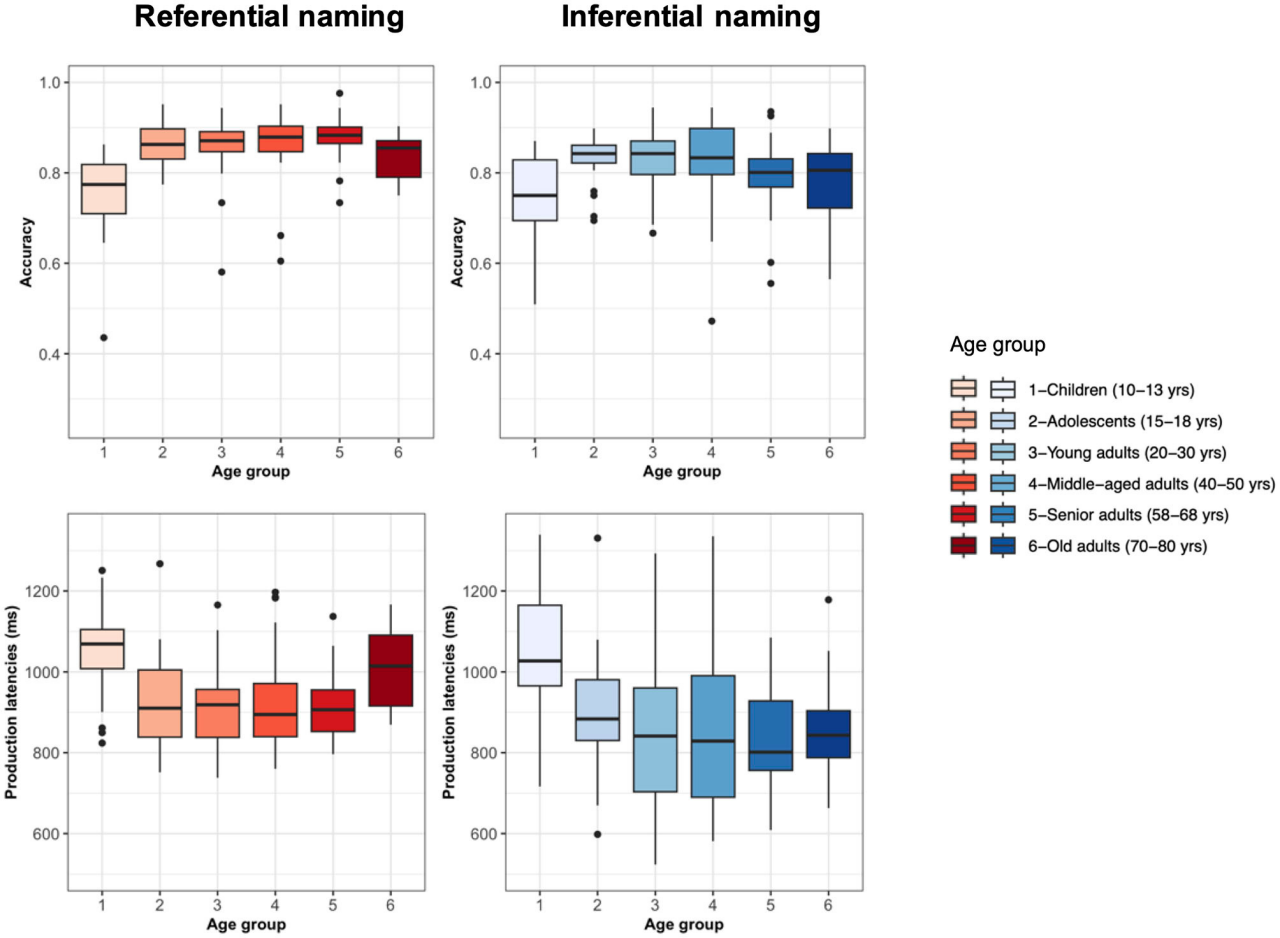
Performance on language production as a function of the six different age groups. Results on accuracy **(top)** and production latencies **(bottom)** are displayed for referential picture naming **(left panels)** and inferential naming from definition **(right panels)**.

Performance in the picture naming task displayed bow-shape and U-shape patterns for accuracy and production latencies, respectively, with lower accuracy and slower naming speed in children and old adults relative to adolescents and young and middle-aged adults (see Figure 1). These latter groups presented the highest accuracy and the shorter production latencies.

ANOVAs indicated an effect of age group on accuracy [F(5,124) = 8.50, *p* < 0.001] and on production latencies [F(5,124) = 5.09, *p* < 0.001] with mainly significant differences between children and the other groups (see Appendices 1–4). The group of old adults also differed significantly from young adults and adolescents. There was no difference, however, between children and old adults in production latencies, and no major difference was found between adolescents, young adults, and middle-aged adults.

Overall, the same bow-shape pattern was seen for accuracy in naming from definition but the U-shape pattern was less pronounced for production latencies, with children demonstrating the slowest naming latencies while an apparent stable performance appears from adolescents to older adults (Figure 1, bottom). Statistical analyses revealed significant effects of age group on accuracy [F(5,124) = 2.91, *p* = 0.016] and production latencies [F(5,124) = 4.98, *p* < 0.001]. Production latencies were significantly slower in children compared to all the other groups including old adults (see Appendix 5 for a summary of accuracy and production latencies across groups).

To further characterize differences in the pattern of age effects in picture naming and naming from definition, because of the non-linearity aspect of the effects, we performed statistical analyses with age as a continuous variable. For picture naming, we found a significant effect of age on accuracy [linear effect (*t* = 2.61, *p* < 0.01) and quadratic effect (*t* = −3.65, *p* < 0.001)] and production latencies [quadratic effect (*t* = 4.01, *p* < 0.001)]. For naming from definition, we found a significant effect of age on accuracy [quadratic effect (*t* = –3.07, *p* = 0.003)] and production latencies [linear effect (*t* = –3.18, *p* = 0.002), quadratic effect (*t* = 2.97, *p* = 0.004)].^2^

Finally, we applied non-parametric multivariate adaptative regression splines via the model MARS (with package Earth, R) to achieve a better approximation of the data than allowed by standard linear models. The MARS model constructs a piecewise linear curve, that is comprised of splines joined by hinges, called knot points. MARS selects the optimal number of knot points by a stepwise selection that is used to minimize the generalized cross-validation error of the model (GCVE). Here, the model MARS was used to identify the number of knot points and their location throughout the lifespan.

This analysis delivered two knots in picture naming: at 21 years and 69 years for accuracy, and at 22 years and 61 years for production latencies. By contrast, only one knot was found in naming from definition: at 21 years for accuracy and 22 years for production latencies.

**Figure 2:**
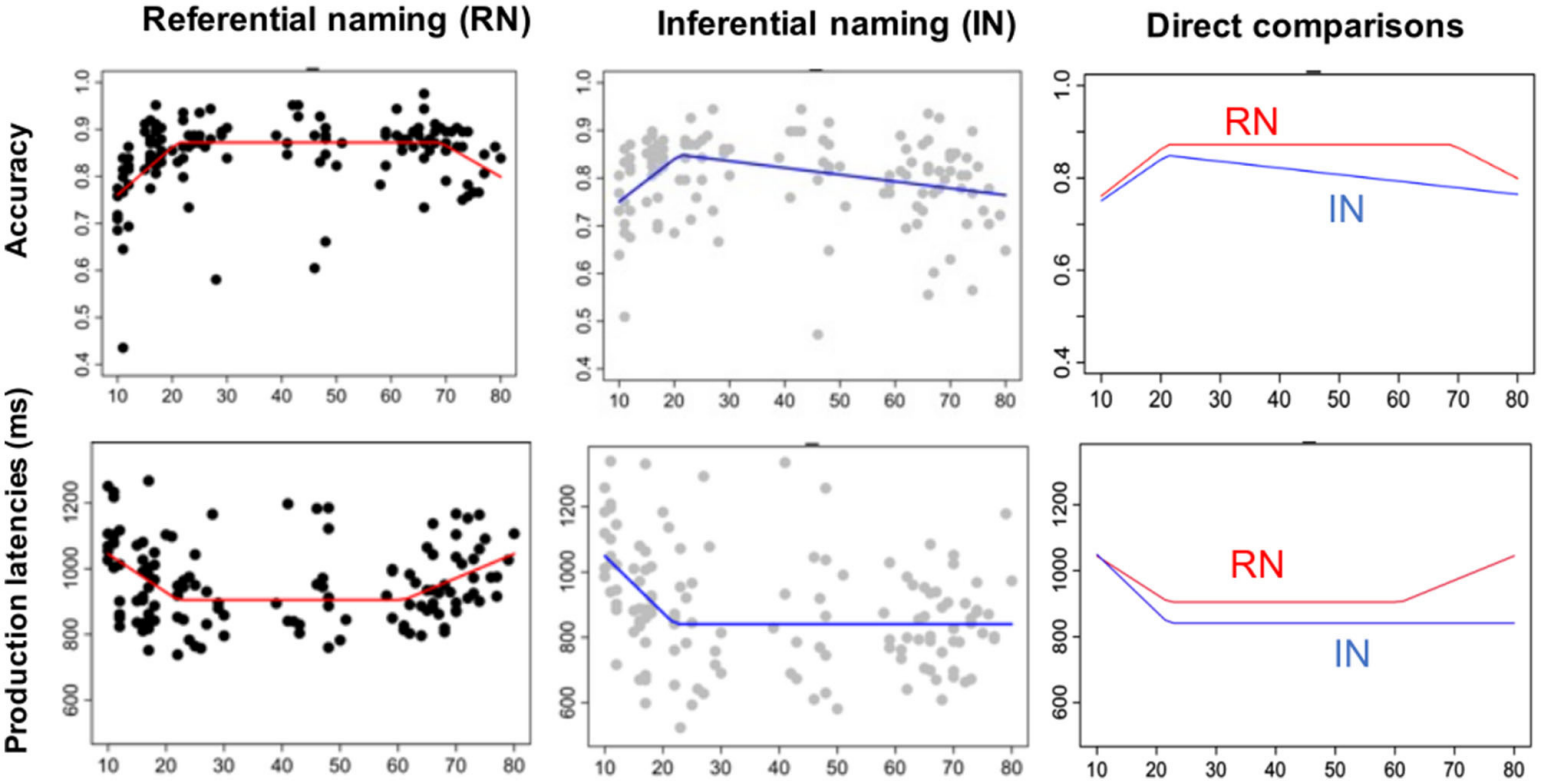
Lifespan patterns of performance on accuracy and production latencies in both language production tasks, with age as a continuous variable. **(Left panel)**: Results obtained for referential picture naming with black dots representing individual data and red lines indicating the multivariate adaptative regression splines. **(Middle panel)**: Results obtained for inferential naming with gray dots representing individual data and blue lines indicating the multivariate adaptative regression splines. **(Right panel)**: Layering of adaptative regression splines obtained for referential naming (in red) and inferential naming (in blue) on accuracy **(top)** and production latencies **(bottom)**. Note that two inflection points/knots are seen for referential naming and only one inflection point/knot for inferential naming.

Figure 2 contrasts these results for picture naming and naming from definition and illustrates the different patterns, with a performance that followed a bow-shape and U-shape curve in picture naming but which was less evident in naming from definition.

### The effect of cognitive measures on picture naming and naming from definition

The analyses in the previous section enabled us to describe different patterns of responses in the two overt naming tasks. In the following, we sought to find predictors of performance across the lifespan by applying multiple regression analyses with cognitive measures obtained during the neuropsychological assessment.

As a pre-requisite, given the large number of independent variables, analyses of correlations were conducted to prevent multicollinearity in multiple regression analyses and determine which factors to consider in the models; given the distribution of performance across ages, we assumed a quadratic relationship with age and applied polynomials by default. The independent variables used as predictors in the models included age as a continuous variable, vocabulary (standard score), digit span (standard score), semantic and phonemic fluencies (number of items), and processing speed (single reaction time task in ms). The tolerance of the models was checked with the VIF function (Field et al., 2012). All VIFs were below 2, which is considered acceptable (Johnston et al., 2018).

For accuracy in picture naming, we observed significant effects of age, semantic fluency, and processing speed (see Table 1). In this model, polynomials were tested for all variables and a quadratic relationship was assumed. Based on the output of the model, we selected linear or quadratic relationships for all variables and ran another model, tested against the first one (when both quadratic and linear effects were found, the polynomial was kept). This second model did not outperform the default model (R^2^ default model = 0.21, R^2^ second model = 0.23, F < 1), although explained variance slightly increased.

**Table 1:** Summary of mixed-effect regression models run for each task on accuracy.

	**Linear**				**Quadratic**			
	**β**	**SE**	***t***	***p***	**β**	**SE**	***t***	***p***
**Picture naming**								
Age	0.31	0.08	3.70	< 0.001	−0.22	0.08	−2.76	0.007
Semantic fluency	0.18	0.08	2.26	0.026	0.05	0.07	0.68	0.499
Processing speed	−0.22	0.08	−2.57	0.011	−0.07	0.07	−1.02	0.309
**Naming from definition**								
Age	0.13	0.08	1.50	0.137	−0.25	0.08	−3.11	0.002
Vocabulary	0.28	0.08	3.67	< 0.001	−0.01	0.07	−0.17	0.864
Working memory	0.17	0.08	2.17	0.032	−0.04	0.07	−0.60	0.550
Semantic fluency	0.20	0.08	2.45	0.016	0.09	0.07	1.27	0.205
Processing speed	−0.33	0.08	−3.88	< 0.001	−0.06	0.07	−0.77	0.441

Only variables that are at least marginally significant (< 0.1) are kept in the table. For complete tables, see Appendix 6.

For accuracy in naming from definition, we found effects of age, vocabulary, digit span, semantic fluency, and processing speed (see Table 1). These results indicated better accuracy in word retrieval from definitions when participants have high vocabulary, digit span, and semantic fluency performance and worse accuracy with increased age and slower processing speed. This model was not outperformed by a model involving linear or quadratic relationships (R^2^ default model = 0.364, R^2^ simpler model = 0.375, F < 1), although the latter had slightly increased explained variance.

Age, semantic fluency, and processing speed were significant predictors of production latencies in referential picture naming (see Table 2). Again, the more complex model was not outperformed by a simpler model [both R^2^ = 0.29, F(1,117) = 1.26, *p* = 0.28]. Overall, the results indicated that performance both in terms of accuracy and speed in picture naming is better when participants have great semantic fluency abilities and worse with increased age and slower processing speed. For production latencies in inferential naming, we found significant effects of age, semantic fluency, and processing speed, as well as a marginal effect of phonemic fluency (see Table 2). The complex model with both linear and quadratic variables was not significantly different than a simpler model (R^2^ default model = 0.35, R^2^ simpler model = 0.37, F < 1), although the latter had slightly increased explained variance.

**Table 2:** Summary of mixed-effect regression model run for each task on production latencies.

	**Linear**				**Quadratic**			
	**β**	**SE**	***t***	***p***	**β**	**SE**	***t***	***p***
**Picture naming**								
Age	−308	123	−2.51	0.013	257	118	2.17	0.032
Semantic fluency	−434	118	−3.67	< 0.001	145	106	1.37	0.172
Processing speed	459	124	3.72	< 0.001	−133	107	−1.24	0.218
**Naming from definition**								
Age	−860	171	−5.04	< 0.001	92	165	0.56	0.576
Semantic fluency	−583	165	−3.54	0.001	−42	147	−0.29	0.774
Phonological fluency	−291	172	−1.69	0.093	75	150	0.50	0.619
Processing speed	699	172	4.06	< 0.001	−193	149	−1.30	0.196

Only variables that are at least marginally significant (< 0.1) are kept in the table. For complete tables, see Appendix 6.

### Analyses with processing speed taken into account

Because processing speed has a great impact on production latencies and to ensure that this impact may not be orthogonal to other measures, we corrected production latencies by processing speed. This correction consisted of an intra-individual normalization of production latencies in picture naming and in naming from definition (RTnaming) from the processing speed measure (RTspeed) under the following form:

$$\begin{array}{l} cL\;=\frac{\text{RTnaming }-\text{RTspeed}}{\text{RTspeed}} \end{array}$$

**Figure 3:**
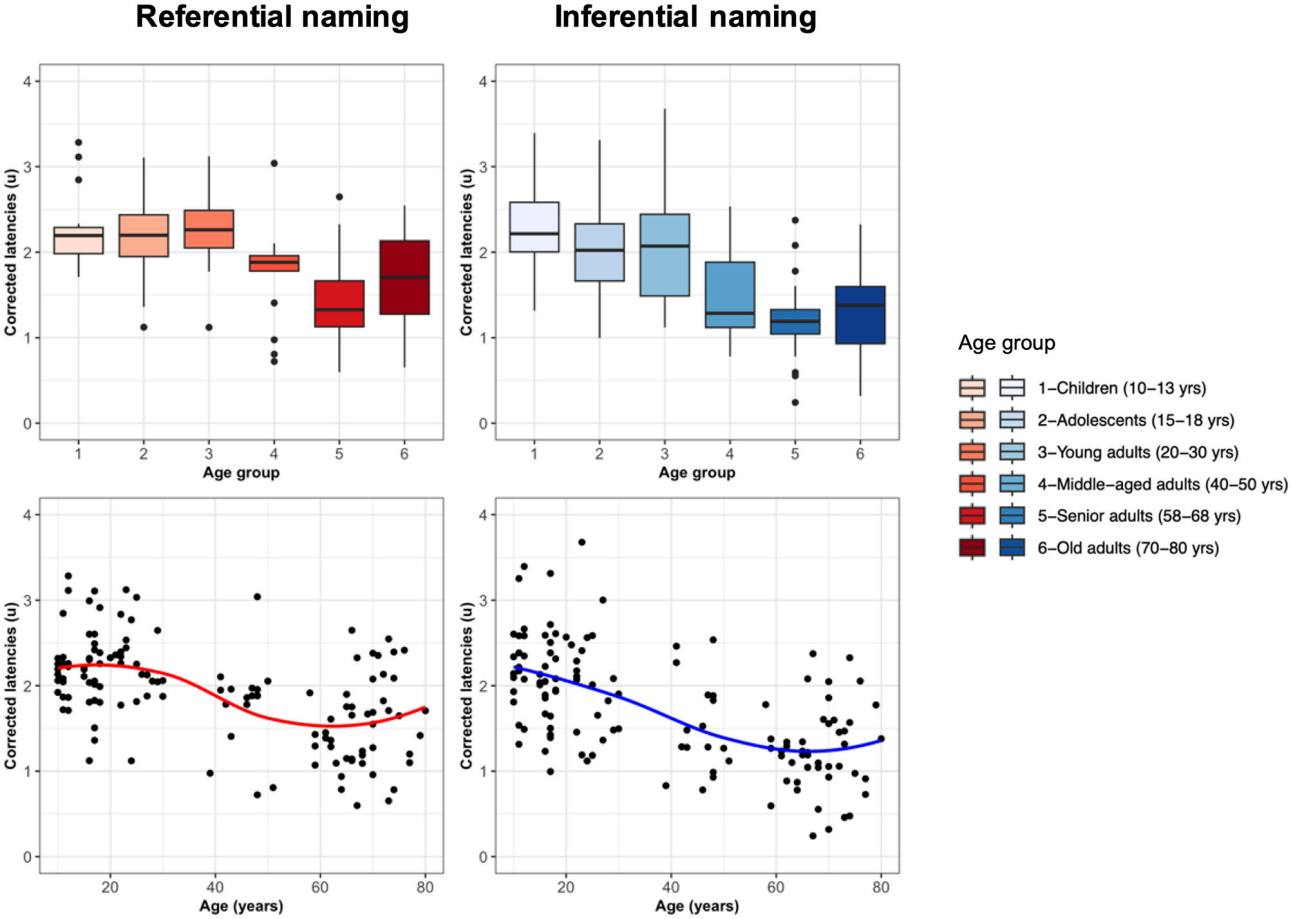
Corrected Latency across age groups **(top panels)** and across individuals as a function of age **(bottom panels)** for referential naming and inferential naming.

The results obtained with this new variable called corrected Latency (cL) reveal similar latencies in young individuals up to 30 years of age when corrected for processing speed (see Figure 3). A decrease in production latencies independent of the processing speed was seen across adulthood but not in the oldest individuals, in particular, for picture naming.

Corrected Latency (cL) was then entered as a dependent variable in a multiple regression model that included continuous age, vocabulary, digit span, and semantic and phonemic fluencies as independent (linear) variables.

Analyses revealed only a significant effect of age (B = −0.01, *t* = –6.19, *p* < 0.001) on picture naming corrected production latencies. For corrected production latencies in naming from definition, a significant effect of age was observed (B = −0.02, *t* = –8.06, *p* < 0.001), as well as an effect of semantic fluency (B = −0.01, *t* = –2.02, *p* = 0.046).

## Discussion

In this study, we used a multivariate approach to describe the lifespan patterns of language production and investigate their explanatory cognitive factors in two experimental tasks involving different elicitation of word production.

### Summary of the results

Our main result is that the patterns of performance observed from 10 years to above 70 years of age are different depending on the language production tasks and the measures, especially in the second half of the lifespan.

At first glance, a similar bow-shape pattern is seen for accuracy in picture naming and naming from definition. Accuracy appears to be greater in young and middle-aged adults (roughly from 20 to 70 years of age) compared to children, teenagers, and older adults. However, a more precise assessment of these patterns using non-parametric adaptative regression suggests that accuracy in naming from definition slightly decreases from 20 years of age onwards, while in picture naming, accuracy is maintained until ~70 years of age, when it starts to decrease. For production latencies, between-task discrepancies are more striking in the second half of the lifespan. For picture naming, we found a U-shape pattern with longer production latencies in children and older adults compared to young adults, whereas for naming from definition, production latencies decrease from children to adolescents and adults, and then maintain a similar speed toward adulthood and late adulthood. In general, we found similar effects of age, fluency, and processing speed in both tasks, but an additional effect of vocabulary and digit span (working memory) in naming from definition. Finally, by correcting latencies for processing speed, we still found an age-related decrease in performance in both tasks, with an additional effect of semantic fluency only for naming from definition.

In the end, our main results are that the course of performance is similar from childhood to adulthood for the two tasks and that the patterns become different in the second half of the lifespan across tasks, likely reflecting the specificities of referential and inferential production and their relationship with aging.

### Age-related changes in language production

First of all, the patterns are very much similar in both tasks from childhood to (young) adulthood, with a gradual increase in performance. This increase in performance is reflected by better accuracy and faster production latencies. The continuous improvement of the ability to produce words is probably underlined by the progressive maturation of neurocognitive processes (see Atanasova et al., 2020 for example in referential naming) and a gradual increase in lexical knowledge.

By contrast, in adulthood, different evolutions for picture naming and naming from definition are observed. The lifespan pattern observed for picture naming follows a U-shape trend which replicates what has been reported in the past (D'Amico et al., 2001; Newman and German, 2005; Verhaegen and Poncelet, 2013) and mimicking what is found in other cognitive domains. Most knowledge on aging in language production comes from referential tasks (Burke and Shafto, 2004) with a major role in visual-conceptual processing. It is also well-known that visual-conceptual processing is particularly impacted by aging, for example, face perception and discrimination (Nakamura et al., 2001; Rousselet et al., 2009), processing checkerboards (Price et al., 2017), and visuo-spatial working memory (Kumar and Priyadarshi, 2013; D'Antuono et al., 2020). Visually evoked neural responses show age-related changes, mostly delays in neural peak latencies in old individuals (see Price et al., 2017). Recent observations suggest that such delays are not necessarily linked to behavioral impairments, but that variations in the ability to mentally represent visual stimuli affect cognitive performance across the lifespan, in particular, object naming in older individuals (Bruffaerts et al., 2019). The observation that inferential naming (tested with a naming from auditory definition task, i.e., exempt of visual-conceptual processing) shows less decrease of performance in older individuals is coherent with the idea that visual-conceptual processing explains referential picture naming performance in aging.

Indeed, performance in naming from definition displays a different pattern across the second half of the lifespan. This is further evidenced by the observation that between-task correlations decrease with age (see footnote in the Results section). Older individuals do not show similar decremental behavior, particularly in production latencies. It should be acknowledged that in naming from definition, we found additional predictors of performance with vocabulary and digit span with respect to picture naming and that individuals with greater semantic fluency were better at word retrieval (i.e., more accurate and faster). Given that the pattern of naming from definition does not suggest decline, it may reflect that different processes are at play in inferential production, possibly making space for compensatory mechanisms. One may argue that the observed differences between picture naming and naming from definition relate more to a difference between visual and auditory processing. This could not explain the results obtained by Fargier and Laganaro (2017) as differences in neural correlates of picture naming and naming from definition were found even though both tasks were delivered through visual inputs. In the present study, however, we introduced this confound for particular reasons: The use of auditory material for the naming from definition task was decided purposely to avoid lifespan differences specifically due to reading abilities/expertise in children. We thus cannot exclude the contribution of these different sensory input processes to the observed effects, in particular, specific decrements in visuo-perceptual or visuo-conceptual processes affecting performance in picture naming. Further work is needed to compare picture naming and inferential naming from definition with visual material. In the next section, we further examine differences between inferential and referential naming that may contribute to explaining these results.

### Comparing word retrieval in inferential naming to referential naming

#### Lexical selection

Despite a continuously growing vocabulary across the lifespan, accuracy in naming from definition slightly decreases. With regard to language production in older individuals, it has been suggested that difficulties could be related to different semantic associations (Wierenga et al., 2008) or to retrieval abilities, which involve semantic, cognitive, and control processes leading to lexical selection (Braver and Barch, 2002), or inefficient phonological word form retrieval (Burke and Mackay, 1997).

As vocabulary increases, target words for definitions might have increased alternates across the lifespan; hence, the slight continuous decrease in accuracy for naming from definition could reflect alternative responses rather than increasing errors. Yet, one may ask whether this is a question of alternates or competitors, that is, a question of the size of the lexicon or of the ability to select target words from that lexicon. One important aspect of theoretical models of lexical access and language production is lexical selection (Caramazza, 1997; Levelt, 1999) where the most appropriate word must be singled out from alternative candidates. In naming from definition, just like in picture naming, several lexical candidates are likely to be activated in response to input. Selection of the target word, and elimination of inappropriate candidates, must take place, while information in the definition is gathered. The lexical selection must be grounded in more general response selection abilities and cognitive control theories (Roelofs, 2003) such as information processing (Cohen et al., 1990), conflict monitoring (Botvinick et al., 2001), and active maintenance of memory representations (Kane and Engle, 2002), which can all be affected by aging (see Braver et al., 2005). Indeed, compared to young individuals, older people show greater sensitivity to interference (Verhaeghen and Cerella, 2002; Manard et al., 2014). This is likely reflected in the reduced accuracy in picture naming across the lifespan, notably in older individuals. Decreased cognitive control abilities, such as being able to remove interfering candidates, are possibly accented in the context of a larger number of candidates in naming from definition. The observed pattern in naming from definition could thus reflect first the effect of increased alternates and then difficulties in reducing interference, the latter also explaining the observed pattern in picture naming in late adulthood. Further work is required to tease apart those hypotheses.

Older healthy people often complain about difficulties in finding their words in connected speech or daily writing, while general knowledge is well-maintained (Beier and Ackerman, 2001). Increased tip-of-the-tongue states, which reflect the inability to produce a known word associated with a strong feeling of knowing, are reported. In those cases, the access to semantics is successful but phonological encoding is often partial (Burke et al., 1991). According to the transmission deficit hypothesis (Burke and Shafto, 2004 for a review), there is a selective weakening of the connections among semantic, lexical, and phonological representations with age, thus explaining reduced accuracy. The aforementioned executive processes and the transmission hypothesis can explain reduced accuracy and slowing down of responses in picture naming. However, in naming from definition, we did not find a similar slowing down of production latencies, which likely calls for compensatory mechanisms in late adulthood. A speculative idea is that there may be differential time and cognitive demand allocated to lexical-semantic and phonological processes in older adults, possibly because of enriched semantic representations, increased vocabulary, and more/less automatic processes. This may explain discrepancies across tasks and needs further investigation. The progressive selection of a word based on associations and knowledge in older adults may also contribute to pre-activate phonological representations, hence leading to a different pattern of results than with picture naming.

#### Lexical prediction

Another feature of word retrieval in inferential naming relative to referential picture naming is the potential role of lexical prediction. Because definitions are provided in a serial fashion, be it in small chunks for written definitions (see Fargier and Laganaro, 2017) or through an auditory stream as here, individuals may apply prediction strategies as the definition unfolds. We can speculate that when prediction is invalidated by the end of the definition, it may lead to incorrect answers or increased production latencies as individuals would need to reevaluate the definition and the lexical candidates. By contrast, if prediction is not invalidated, inappropriate candidates may have been eliminated early and the target word may have been provided faster. Insights from comprehension studies suggest that lexical prediction could actually explain the specific pattern observed in naming from definition in late adulthood. In their sentence reading study, Rayner and collaborators (2006) found that older readers skip words more often than younger readers and make more regressions back to words. Prediction of upcoming information is based on contextual information and also on general semantic knowledge (Altmann and Mirković, 2009; Hagoort and Indefrey, 2014; Huettig, 2015; Kuperberg and Jaeger, 2016). As a result, when reading or hearing a sentence, comprehenders pre-activate likely upcoming words and their corresponding semantic features. This would facilitate word retrieval in naming from definition task, where comprehension meets production. Older individuals may engage more predictive processes in naming from definition, possibly to compensate for other altered cognitive processes (Rayner et al., 2006; Huettig and Janse, 2016; Kukona et al., 2016). This idea is supported by the fact that older individuals often exhibit greater knowledge and that general comprehension mechanisms are well-preserved in aging (see Shafto and Tyler, 2014). Although the neural mechanisms underlying such predictive processes are assumed to be modulated in older adults (Wlotko et al., 2012), a recent study showed preserved behavioral facilitation for successful predictions in older adults (Dave et al., 2018). For instance, older adults with greater verbal fluency display similar neural correlates as younger individuals in tasks involving lexical prediction (see Federmeier et al., 2010; DeLong et al., 2012). Dave and collaborators (2018) did not find evidence that greater verbal fluency correlates with better prediction accuracy, but nonetheless argued that older individuals who display greater verbal fluency are likely to engage more in predictive mechanisms. In the present study, we found a significant effect of semantic fluency on accuracy and production latencies, such that individuals with greater fluency performed better in the naming from definition. Recall that we assessed verbal fluencies because it has been suggested that lexical prediction was related to those abilities, in particular, semantic fluency, which requires managing and inhibiting semantic competitors (Thompson-Schill et al., 1997). However, we acknowledge that more work is needed to highlight the contribution of lexical prediction to inferential word retrieval and determine to what extent the maintenance of performance in naming from definition in late adulthood is due to preserved, or even enhanced, prediction abilities in old individuals.

### Other cognitive components

We also reported an effect of digit span on inferential naming accuracy. The relationship between working memory and sentence processing/comprehension has been the focus of many studies, notably concerning its role in maintaining sentence constituents before integration and maintaining predictions (DeDe et al., 2004; Waters and Caplan, 2004; Fedorenko et al., 2007). In the present study, individuals who display this relatively well-preserved ability are likely to be better at inferential naming, even in old adults. This result is in line with a recent study with aphasic participants showing that greater short-term memory capacities are associated with fewer errors (Sayers et al., 2023). These observations highlight the link between the successful activation of linguistic representations and temporary storage and maintenance of information, which seems particularly important in naming from definition.

Studies often use the picture naming task to elicit word production, and our study gives an idea of the expected performance at any age between 10 and 80 years in both picture naming and naming from definition. Although intuitive, slowing down production latencies across the lifespan cannot be reduced to differences in processing speed. To investigate this specific aspect beyond regression analyses, we corrected production latencies by normalizing them with performance in another reaction time task to remove the part of the performance that is explicitly related to processing differences. If naming performance was exclusively dependent on speed, we would not find an effect of age on corrected latencies. We found an effect of age on production latencies in both tasks, indicating that the decline is not accounted for solely by reduced speed. However, we also find an effect of semantic fluency on corrected latencies in naming from definition only, suggesting that performance in this task is more related to semantic control than performance in picture naming. This strengthens the idea that both tasks do not rely on the same cognitive abilities or to a different extent.

## Limitations

This study is one of the few that investigates language processing across the lifespan, spanning childhood to late adulthood, but some limitations are worth mentioning. Although we collected data from more than 140 participants, which represent a high number considering that they also engaged in neural recordings, the sample size may seem small considering the range in age. Moreover, although we introduced several cognitive assessments, additional measurements could have been helpful to better characterize aging differences and disentangle cognitive processes across the tasks. For example, because naming from definition was delivered from auditory input and picture naming from visual input, tasks that measure visuo-perceptual processes and auditory processes could have been useful to determine the role of visuo-perceptual abilities in decremental performance in picture naming in older individuals. Moreover, as we hypothesize that maintenance of performance in naming from definition is partly due to predictive processing, tasks that measure this ability (e.g., filled-gap tasks) should be introduced in future work. Finally, although we investigated several cognitive abilities to explain the maintenance or decline of naming abilities across the lifespan, we did not inquire about language experience. Multilingualism can influence several cognitive processes, including executive functions that are involved in word retrieval, but research often offered contradictory findings (Duñabeitia and Carreiras, 2015; Guzmán-Vélez and Tranel, 2015). The effect of language experience on naming abilities across the lifespan, and, in particular, in aging, should be the focus of an entire project (Rossi and Diaz, 2016), and it was beyond the scope of the present study.

## Conclusive remarks

The present study aimed to fill two gaps in the literature. First, most studies investigating language production focus on healthy young individuals or are from compartmentalized studies in childhood or late adulthood. Between-group life-span studies remain scarce, although they are likely to better evidence the dynamic nature of language abilities and to better serve standardization of language performance for clinical purposes. The strength of this study was to compare two tasks (picture naming and naming from definitions) that involve different cognitive processes underlying word production, with the same sample of individuals. The two tasks show both similarities and differences in age-related trajectories and predictors, but the data obtained in naming from definitions challenge our preconceptions about the fate of language production, i.e., maintained performance instead of decreasing ability. Further work may take care to decipher whether all underlying cognitive processes have the same properties, or if not, which mental operations are impaired by cognitive aging and which are subject to compensatory mechanisms. In the end, the present study probably tells us as much about inferential naming as about cognitive aging.

## Data availability statement

The datasets presented in this study can be found in online repositories. The names of the repository/repositories and accession number(s) can be found below: https://osf.io/jwyur/.

## Ethics statement

The studies involving humans were approved by Local Ethics Committee of the Faculty of Psychology and Educational Science of the University of Geneva. The studies were conducted in accordance with the local legislation and institutional requirements. Written informed consent for participation in this study was provided by the participants' legal guardians/next of kin.

## Author contributions

All authors listed have made a substantial, direct, and intellectual contribution to the work and approved it for publication.

## Footnotes

^1^A larger set of definitions (*N* = 144) was pre-tested on 35 undergraduate students aged 19–44 years (average: 23, SD = 5;8 years); items that did not reach the 65% of agreement were not included in this study; thus, from the initial 144 definitions, 31 were withdrawn and 5 were used as warm-up fillers.

^2^Following recommendations of an external reviewer, we conducted between-task correlations for each group, and both for accuracy (ACC) and production latencies (RTs). Correlations were lower for accuracy compared to production latencies and varied across age. In children and adolescents, correlations were lower on accuracy than on production latencies (in children: Acc = 0.63; RT = 0.72; in adolescents: Acc = 0.52; RT = 0.82). Greatest between-task correlations were found in middle-age adults (Acc = 0.87; RT = 0.75) and young adults (Acc = 0.69; RT = 0.78). Between-task correlations were lower in groups of older individuals (in senior adults: Acc = 0.60; RT = 0.68; in old adults: Acc = 0.45; RT = 0.62).
