# Supplementary material for: Referential and inferential production across the lifespan: different patterns and different predictive cognitive factors
Source: Front Psychol. 2023 Oct 30;14:1237523. doi: 10.3389/fpsyg.2023.1237523 (PMC10643179; doi:10.3389/fpsyg.2023.1237523)
Supplement: Supplementary file 1 [file Data_Sheet_1.docx]

**Appendix 1** : Summary of statistical differences across age-groups on picture naming accuracy. Data indicate *t values*. *** < 0.0001 ; ** < 0.001; * < 0.01.

|  | 1-Children | 2-Adolescents | 3-Young adults | 4-Middle age adults | 5-Old adults | 6-Elderly adults |
| --- | --- | --- | --- | --- | --- | --- |
| 1-Children |  | 5.147 *** | 4.951 *** | 4.462 *** | 5.979 *** | 3.940 *** |
| 2-Adolescents |  |  | -0.157 | -0.287 | 0.791 | -1.114 |
| 3-Young adults |  |  |  | -0.141 | 0.942 | -0.952 |
| 4-Middle age adults |  |  |  |  | 1.010 | -0.742 |
| 5-Old adults |  |  |  |  |  | 1.899 |
| 6-Elderly adults |  |  |  |  |  |  |

**Appendix 2** : Summary of statistical differences across age-groups on naming from definition accuracy. Data indicate *t values*. *** < 0.0001 ; ** < 0.001; * < 0.01.

|  | 1-Children | 2-Adolescents | 3-Young adults | 4-Middle age adults | 5-Old adults | 6-Elderly adults |
| --- | --- | --- | --- | --- | --- | --- |
| 1-Children |  | 3.084 ** | 2.865 ** | 2.310 * | 1.559 | 0.904 |
| 2-Adolescents |  |  | -0.201 | -0.554 | -1.682 | -2.211 * |
| 3-Young adults |  |  |  | -0.366 | -1.459 | -1.995 * |
| 4-Middle age adults |  |  |  |  | -0.964 | -1.487 |
| 5-Old adults |  |  |  |  |  | 0.629 |
| 6-Elderly adults |  |  |  |  |  |  |

**Appendix 3** : Summary of statistical differences across age-groups on picture naming latencies. Data indicate *t values*. *** < 0.0001 ; ** < 0.001; * < 0.01.

|  | 1-Children | 2-Adolescents | 3-Young adults | 4-Middle age adults | 5-Old adults | 6-Elderly adults |
| --- | --- | --- | --- | --- | --- | --- |
| 1-Children |  | -3.275 ** | -3.905 *** | -2.913 ** | -3.927 *** | -1.132 |
| 2-Adolescents |  |  | -0.702 | 0.104 | -0.634 | 2.166 |
| 3-Young adults |  |  |  | 0.744 | 0.088 | 2.823 ** |
| 4-Middle age adults |  |  |  |  | -0.682 | 1.882 |
| 5-Old adults |  |  |  |  |  | 2.818 ** |
| 6-Elderly adults |  |  |  |  |  |  |

**Appendix 4** : Summary of statistical differences across age-groups on naming from definition latencies. Data indicate *t values*. *** < 0.0001 ; ** < 0.001; * < 0.01.

|  | 1-Children | 2-Adolescents | 3-Young adults | 4-Middle age adults | 5-Old adults | 6-Elderly adults |
| --- | --- | --- | --- | --- | --- | --- |
| 1-Children |  | -3.253 ** | -3.980 *** | -3.460 *** | -4.506 *** | -3.828 *** |
| 2-Adolescents |  |  | -0.806 | -0.493 | -1.276 | -0.714 |
| 3-Young adults |  |  |  | 0.247 | -0.440 | 0.072 |
| 4-Middle age adults |  |  |  |  | -0.657 | -0.175 |
| 5-Old adults |  |  |  |  |  | 0.504 |
| 6-Elderly adults |  |  |  |  |  |  |

**Appendix 5** : Summary of accuracy and production latencies for referential naming (picture naming) and inferential naming (naming from definition). Mean (SD) are presented for each group of participants.

|  |  | **Referential naming** | |  | **Inferential naming** | |
| --- | --- | --- | --- | --- | --- | --- |
| **Age group** |  | *Accuracy* | *Production latencies* |  | *Accuracy* | *Production latencies* |
| *1-Children (10-13 yrs)* |  | 0.75 (0.1) | 1048 (123) |  | 0.75 (0.1) | 1054 (150) |
| *2-Adolescents (15-18 yrs)* |  | 0.86 (0.04) | 934 (116) |  | 0.83 (0.05) | 890 (156) |
| *3-Young Adults (20-30 yrs)* |  | 0.86 (0.08) | 911 (114) |  | 0.83 (0.07) | 851 (202) |
| *4-Middle-aged Adults (40-50 yrs)* |  | 0.86 (0.09) | 938 (146) |  | 0.82 (0.1) | 864 (218) |
| *5-Old Adults (58-68 yrs)* |  | 0.88 (0.05) | 914 (87) |  | 0.79 (0.09) | 831 (122) |
| *6-Elderly Adults (70-80 yrs)* |  | 0.84 (0.05) | 1008 (97) |  | 0.77 (0.09) | 855 (132) |

**Appendix 6** : Complete summaries of mixed-effect regression models run for each task on accuracy (top table) and on production latencies (bottom table).

Model formula:

lm(DV~poly(Age,2)+poly(Vocabulary,2)+poly(Working memory,2)+poly(Semantic fluency,2)+poly(Phonological fluency,2)+poly(Processing speed,2), data)

Accuracy

|  | Linear | | | |  | Quadratic | | | |
| --- | --- | --- | --- | --- | --- | --- | --- | --- | --- |
|  | 𝛃 | SE | t | *p* |  | 𝛃 | SE | t | *p* |
| **Picture naming** |  |  |  |  |  |  |  |  |  |
| Age | 0.31 | 0.08 | 3.70 | <0.001 |  | -0.22 | 0.08 | -2.76 | 0.007 |
| Vocabulary | 0.11 | 0.08 | 1.45 | 0.150 |  | 0.03 | 0.07 | 0.44 | 0.662 |
| Working memory | 0.01 | 0.08 | 0.17 | 0.866 |  | -0.01 | 0.07 | -0.15 | 0.878 |
| Semantic fluency | 0.18 | 0.08 | 2.26 | 0.026 |  | 0.05 | 0.07 | 0.68 | 0.499 |
| Phonological fluency | -0.04 | 0.08 | -0.48 | 0.631 |  | 0.01 | 0.07 | 0.16 | 0.877 |
| Processing speed | -0.22 | 0.08 | -2.57 | 0.011 |  | -0.07 | 0.07 | -1.02 | 0.309 |
| **Naming from definition** |  |  |  |  |  |  |  |  |  |
| Age | 0.13 | 0.08 | 1.50 | 0.137 |  | -0.25 | 0.08 | -3.11 | 0.002 |
| Vocabulary | 0.28 | 0.08 | 3.67 | <0.001 |  | -0.01 | 0.07 | -0.17 | 0.864 |
| Working memory | 0.17 | 0.08 | 2.17 | 0.032 |  | -0.04 | 0.07 | -0.60 | 0.550 |
| Semantic fluency | 0.20 | 0.08 | 2.45 | 0.016 |  | 0.09 | 0.07 | 1.27 | 0.205 |
| Phonological fluency | -0.11 | 0.08 | -1.27 | 0.206 |  | 0.01 | 0.07 | 0.14 | 0.889 |
| Processing speed | -0.33 | 0.08 | -3.88 | 0.000 |  | -0.06 | 0.07 | -0.77 | 0.441 |

Production latencies

|  | Linear | | | |  | Quadratic | | | |
| --- | --- | --- | --- | --- | --- | --- | --- | --- | --- |
|  | 𝛃 | SE | t | *p* |  | 𝛃 | SE | t | *P* |
| **Picture naming** |  |  |  |  |  |  |  |  |  |
| Age | -308 | 123 | -2.51 | 0.013 |  | 257 | 118 | 2.17 | 0.032 |
| Vocabulary | 58 | 111 | 0.52 | 0.602 |  | -45 | 106 | -0.43 | 0.670 |
| Working memory | 18 | 117 | 0.16 | 0.876 |  | 141 | 104 | 1.36 | 0.178 |
| Semantic fluency | -434 | 118 | -3.67 | <0.001 |  | 145 | 106 | 1.37 | 0.172 |
| Phonological fluency | -127 | 123 | -1.03 | 0.307 |  | -150 | 108 | -1.40 | 0.165 |
| Processing speed | 459 | 124 | 3.72 | <0.001 |  | -133 | 107 | -1.24 | 0.218 |
| **Naming from definition** |  |  |  |  |  |  |  |  |  |
| Age | -860 | 171 | -5.04 | <0.001 |  | 92 | 165 | 0.56 | 0.576 |
| Vocabulary | -167 | 154 | -1.08 | 0.282 |  | 23 | 147 | 0.16 | 0.877 |
| Working memory | 157 | 162 | 0.97 | 0.335 |  | 38 | 145 | 0.26 | 0.795 |
| Semantic fluency | -583 | 165 | -3.54 | 0.001 |  | -42 | 147 | -0.29 | 0.774 |
| Phonological fluency | -291 | 172 | -1.69 | 0.093 |  | 75 | 150 | 0.50 | 0.619 |
| Processing speed | 699 | 172 | 4.06 | <0.001 |  | -193 | 149 | -1.30 | 0.196 |
